# Increased Anxiety in Offspring Reared by Circadian *Clock* Mutant Mice

**DOI:** 10.1371/journal.pone.0066021

**Published:** 2013-06-12

**Authors:** Hiroko Koizumi, Nobuhiro Kurabayashi, Yuto Watanabe, Kamon Sanada

**Affiliations:** 1 Department of Biophysics and Biochemistry, Graduate School of Science, The University of Tokyo, Tokyo, Japan; 2 Molecular Genetics Research Laboratory, Graduate School of Science, The University of Tokyo, Tokyo, Japan; University of Texas Southwestern Medical Center, United States of America

## Abstract

The maternal care that offspring receive from their mothers early in life influences the offspring’s development of emotional behavior in adulthood. Here we found that offspring reared by circadian clock-impaired mice show elevated anxiety-related behavior. *Clock* mutant mice harboring a mutation in *Clock*, a key component of the molecular circadian clock, display altered daily patterns of nursing behavior that is fragmented during the light period, instead of long bouts of nursing behavior in wild-type mice. Adult wild-type offspring fostered by *Clock* mutant mice exhibit increased anxiety-related behavior. This is coupled with reduced levels of brain serotonin at postnatal day 14, whose homeostasis during the early postnatal period is critical for normal emotional behavior in adulthood. Together, disruption of the circadian clock in mothers has an adverse impact on establishing normal anxiety levels in offspring, which may increase their risk of developing anxiety disorders.

## Introduction

Anxiety is an emotional state that is elicited in anticipation of threat and is essential for organisms to adapt to adverse circumstances. However, excessive or inappropriate anxiety leads to various mental disorders such as anxiety disorders. Human susceptibility to mental disorders such as mood and anxiety disorders can be determined early in life by genetic and environmental factors [1]–[6]. Particularly, maternal behavior has long-lasting effects on emotional behavior of the offspring. In rats, offspring reared by less licking/grooming/arched-back nursing mothers exhibit increased anxiety-related behavior and stress response, compared to offspring reared by high licking/grooming/arched-back nursing mothers [7], [8]. Cross-fostering studies have revealed that these influences are primarily attributable to the difference of the maternal behavior of the mothers [9]. Also, in nonhuman primates and rats, reduced levels of maternal care, such as maternal deprivation and neglect, leads to an increase in anxiety-related behavior of the offspring [10]–[12]. In humans, childhood adversity such as childhood abuse, maternal deprivation and maternal neglect is associated with a significantly increased risk for multiple forms of mental disorders [13]–[16]. Thus, aversive maternal care received during the early development influences negatively the development of normal anxiety-related behavior of the offspring.

In mammals, physiological and behavioral rhythms are generated by a circadian clock located in the suprachiasmatic nucleus of the hypothalamus [17], [18]. The circadian clock comprises transcription/translation-based feedback loops of clock components such as *Clock*, *Bmal1*, *Period* and *Cryptochrome* [17], [18]. CLOCK and BMAL1, two transcription factors, activate the transcription of *Period* and *Cryptochrome* genes, and PERIOD and CRYPTOCHROME proteins in turn suppress their own transcription through negative regulation of CLOCK and BMAL1 to generate a circadian oscillation of the molecular clock [17]–[19]. In rodents, the circadian clock governs nursing behavior that shows a clear diurnal rhythm with higher amount during the light period and lower during the dark period, as mice with an impaired circadian clock display disrupted rhythm of nursing behavior [20]–[22]. This raises the hypothesis that offspring reared by mothers with impaired circadian clock may exhibit emotional disturbance in adulthood, due to the altered maternal care received.

In the present study, we examined levels of anxiety in mice reared by mothers with a mutation in *Clock*, a key component of the molecular circadian clock. We showed that *Clock* mutant mothers display the altered daily pattern of nursing behavior and that maternal care provided by *Clock* mutant mothers predisposes the offspring to increased anxiety-related behavior. Furthermore, the offspring at postnatal day 14 showed reduced levels of brain serotonin that is known to be essential to the establishment of anxiety circuits during the postnatal development. Thus, the present study underscores an adverse impact of circadian-clock disruption in mothers on anxiety levels in the offspring and suggests that appropriate daily patterns of maternal care may be crucial for establishing normal anxiety-related behavior in the offspring.

## Results

### Altered Daily Patterns of Nursing Behavior in *Clock* Mutant Mice

**Figure 1:**
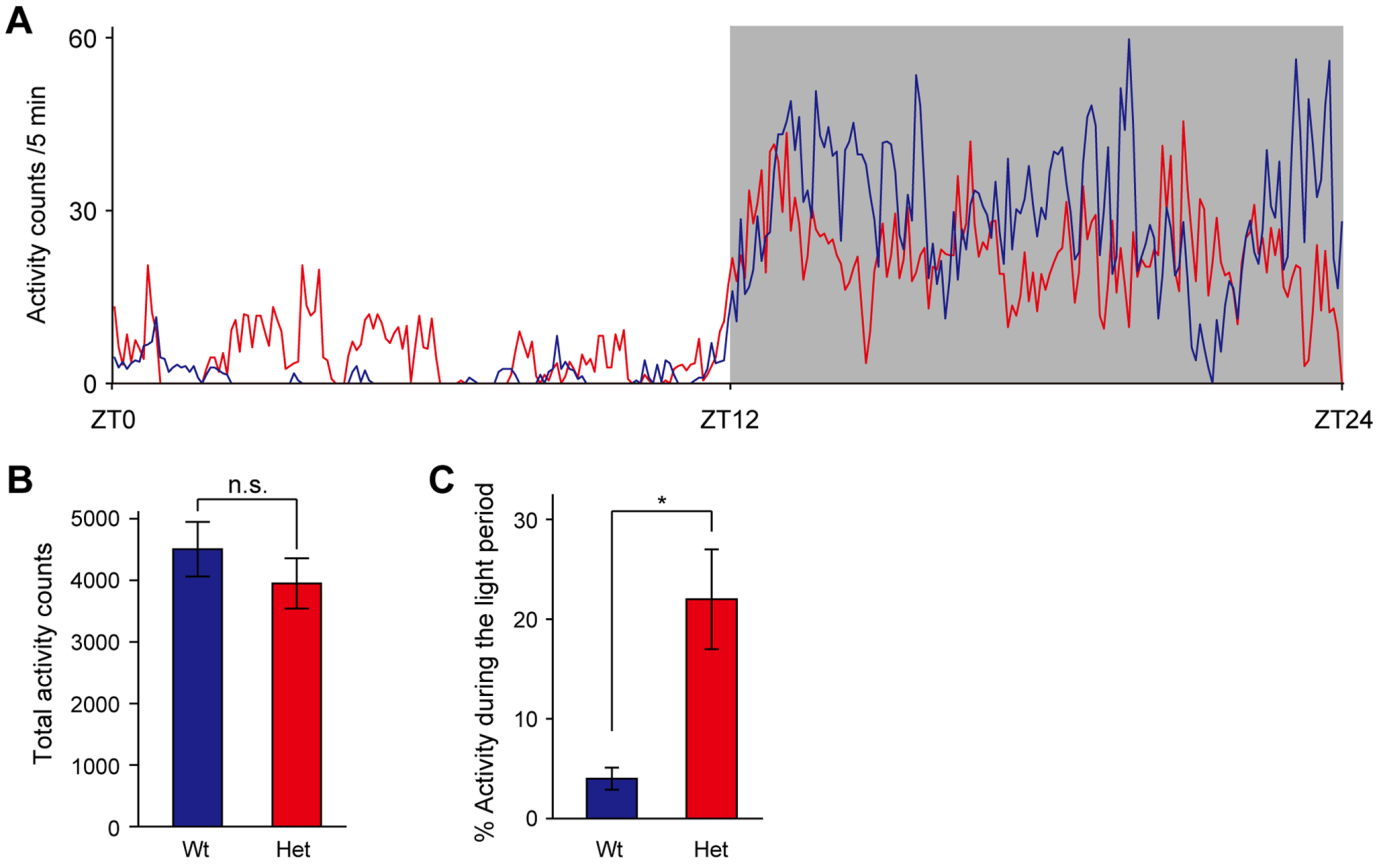
Altered diurnal pattern in locomotor activity of female heterozygous *Clock* mutant mice. (A) Activity counts over the 24-hour cycle during the light (unshaded) and dark (shaded) periods. Mean activity counts in 5-min bins of wild-type mice (blue line, n = 4) and heterozygous *Clock* mutant mice (red line, n = 4) are plotted. (B) Activity counts were accumulated over the 12-hour light and 12-hour dark periods, and total activity counts were presented as mean ± SEM (n = 4). t(6) = 0.924, p>0.05 by Student’s *t* test. n.s.: not significant. (C) Light-period activity counts were expressed as a percentage of total activity counts (n = 3). t(4) = −3.39, *p<0.05 by Student’s *t* test. Wt: wild-type mice, Het: heterozygous *Clock* mutant mice.

Mice harboring a mutation in *Clock* gene, which results in a dominant-negative protein with low transactivation ability, show changes in circadian rhythmicity [23]–[25]. Heterozygous *Clock* mutant mice display a1-hour increase in the period of the free running rhythm of locomotor activity in constant darkness, and homozygous mice exhibit a 3- to 4-hour increase in circadian period, which is often followed by arrhythmicity in constant darkness [25]. In a light-dark cycle, homozygous *Clock* mutant mice show the diurnal rhythm in locomotor activity, but exhibit an abnormal increase in activity during the light phase [26]. Similar to the phenotype of homozygous *Clock* mutant mice, heterozygous *Clock* mutant mice display a profound increase in activity during the light phase without alternation of the total activity levels (Fig. 1A–C). Thus, *Clock* mutant mice display the altered diurnal rhythm in locomotor activity in a light-dark cycle.

**Figure 2:**
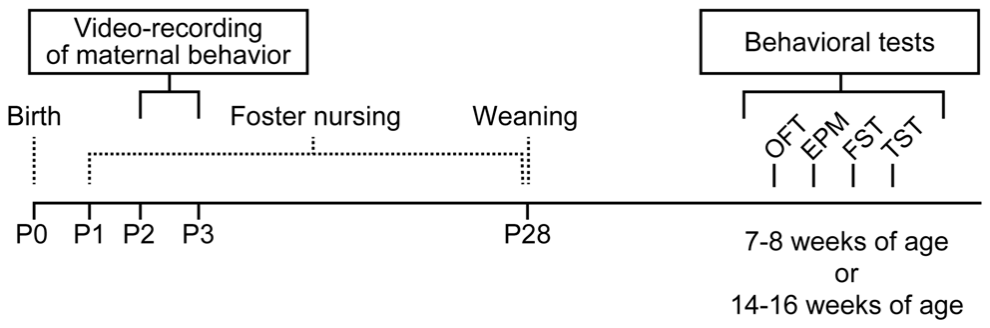
Schematic representation of the experimental design. Wild-type male neonates (postnatal day 1, P1) were separated from their wild-type mother and fostered on postpartum wild-type mice or heterozygous *Clock* mutant mice. At P2-3, maternal behavior of mothers was video-recorded. The offspring were weaned at P28. The offspring at 7–8 weeks of age or at 14–16 weeks of age were subjected to four behavioral tests in the following order; open-field test (OFT), elevated plus maze test (EPM), forced swim test (FST) and tail suspension test (TST). Mice were given one test per day for 4 consecutive days.

**Figure 3:**
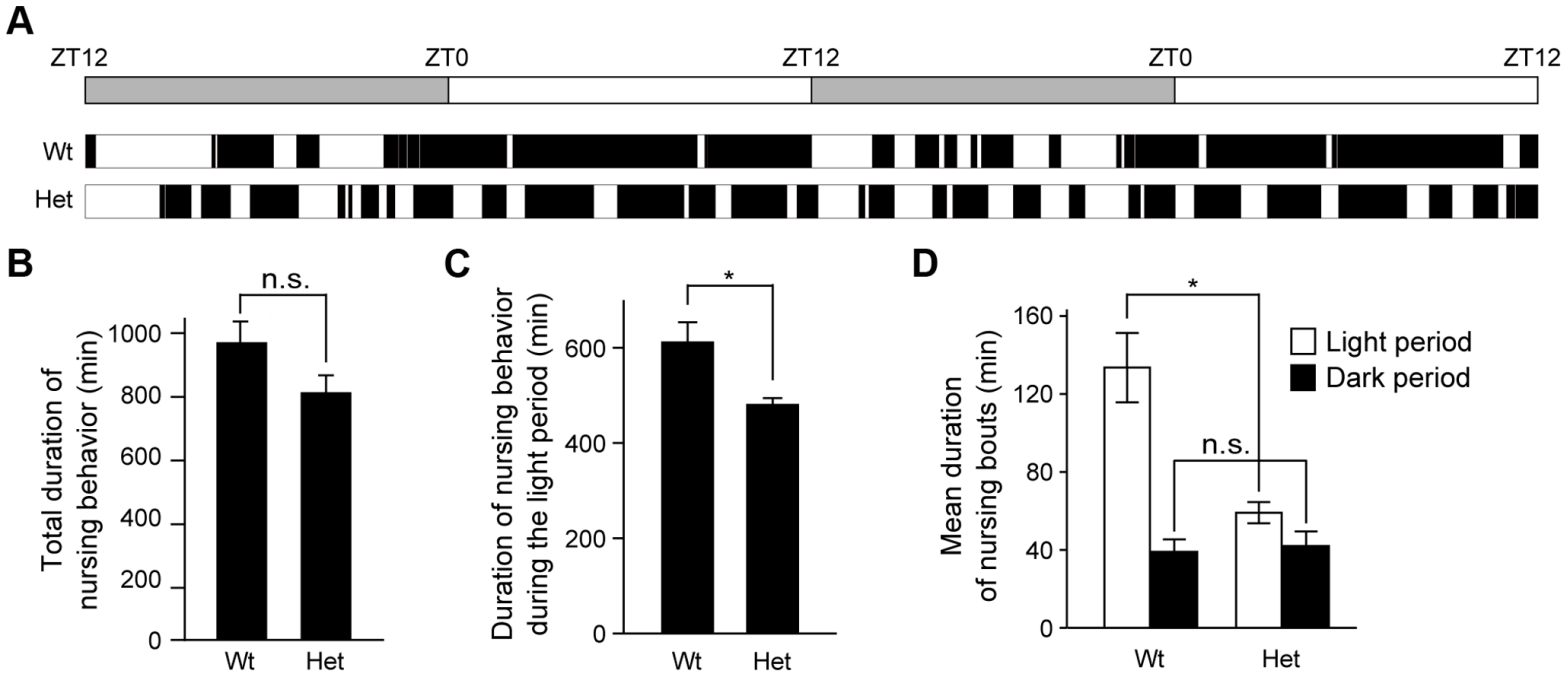
Altered diurnal pattern in nursing behavior of female heterozygous *Clock* mutant mice. (A) Representative actogram of nursing behavior in wild-type and heterozygous *Clock* mutant mice at postpartum day 2–3 under a light-dark cycle (indicated by a bar at top). Black bars represent duration of nursing bouts. (B–D) Total duration of nursing activity per day (B), duration of nursing activity during the light period (C) and mean duration of nursing bouts (D) are shown as mean ± SEM (n = 4, n.s.: not significant, *p<0.05, Student’s *t* test). t(6) = 1.82, p>0.05 in (B), t(6) = 2.93, p<0.05 in (C), t(4) = 4.00, p<0.05 and t(6) = −0.318, p>0.05 in (D). Wt: wild-type mice, Het: heterozygous *Clock* mutant mice.

To explore the influence of *Clock* mutant mothers on the offspring, we used heterozygous *Clock* mutant mice, due to impaired reproductive function and poor milk production of homozygous *Clock* mutant female mice [21], [27]–[29]. We first examined the diurnal pattern of nursing behavior in heterozygous *Clock* mutant mice (referred to hereinafter as *Clock* mutant mice) on postpartum day 2–3 (see Fig. 2). When mother mice exhibit nursing postures (either an arched-back nursing posture, a blanket nursing posture in which mother lays over the pups, or a passive nursing posture in which mother is lying on her side with pups attached) for at least 5 min [21], [30], [31], we measured duration time of the nursing bouts (Fig. 3A). Wild-type mice, on postpartum day 2–3, exhibited nursing bouts that last for a long time (mean duration: 133 min) during the light phase and were short/intermittent (mean duration: 39 min) during the dark phase (Fig. 3D). Total duration of nursing behavior of *Clock* mutant mice within a day was not significantly different from that of wild-type mice (Fig. 3B), whereas *Clock* mutant mice displayed a slight decrease in nursing behavior during the light phase (Fig. 3C). Noticeably, nursing behavior in *Clock* mutant mice was fragmented during the light phase, as evidenced by shorter duration of nursing bouts than that of wild-type mice during the light phase (Fig. 3D). Together, *Clock* mutant mice showed the altered daily pattern of their nursing behavior.

### Elevated Anxiety-related Behavior in Offspring Reared by *Clock* Mutant Mice

**Figure 4:**
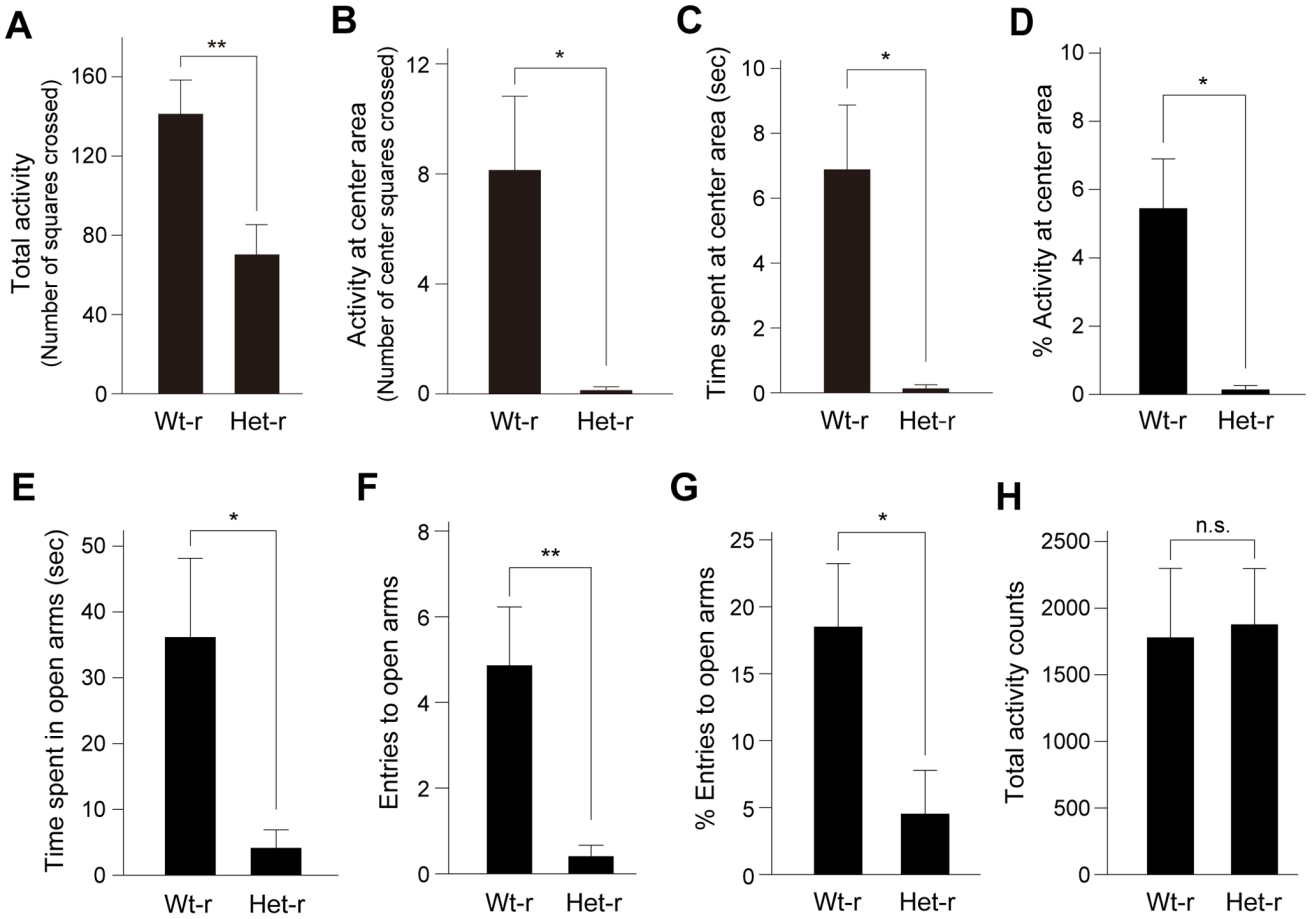
Elevated anxiety-related behavior in offspring reared by *Clock* mutant mice. (A–D) In the open-field test, total activity counts measured by the total number of squares crossed by the mouse (A), activity counts at the center area measured by the number of center squares crossed (B), time spent at the center area (C) and the percentage of the number of center squares crossed (100×center squares/total squares crossed) (D) are shown. Data are presented as mean ± SEM (n = 8, *p<0.05, **p<0.01, Student’s *t* test). t(14) = 3.09, p<0.01 in (A), t(7) = 2.96, p<0.05 in (B), t(7) = 3.38, p<0.05 in (C), t(7) = 3.22, p<0.05 in (D). Wt-r: wild-type mother-reared mice, Het-r: heterozygous *Clock* mutant mother-reared mice. (E–G) In the elevated plus maze test, time spent in the open arms (E), entries to the open arms (F) and the percentage of open arm entries (100×open arm/total entries) (G) are shown. Data are presented as the mean ± SEM (n = 10–14, *p<0.05, **p<0.01, Student’s *t* test). t(14) = 2.60, p<0.05 in (E), t(14) = 3.19, p<0.01 in (F), t(22) = 2.21, p<0.05 in (G). (H) Home cage activity was measured, and total activity counts over the 24-hour cycle are presented as mean ± SEM (n = 3). t(4) = −0.146, p>0.05 by Student’s *t* test. n.s.: not significant.

To examine the influence of heterozygous *Clock* mutant mothers on behavioral phenotypes of the offspring, wild-type postnatal day 1 neonates (6–8 male neonates) were subjected to foster nursing by either wild-type or *Clock* mutant mice. Body weight of pups (postnatal day 31) reared by *Clock* mutant mice did not significantly differ from those reared by wild-type mice (wild-type-reared pups: 14.1 g ±0.34 g, n = 8; *Clock* mutant-reared pups: 13.7 g ±0.55 g, n = 16, p = 0.59, Student’s *t* test), suggesting that growth of pups reared by *Clock* mutant mice was almost normal. The offspring were weaned at 28 days old and then subjected to behavioral tests at 7–8 weeks of age (Fig. 2). We then measured anxiety-related behavior by using open field test (OFT) and elevated plus maze test (EPM), in which the amount of time spent in an anxiety-provoking space such as the center area of the open field or unprotected arms of the raised platform was used as a measure. In the OFT, the offspring showed significantly decreased total activity, spent less time at the center area and less frequently entered the center area (Fig. 4A–D). The offspring also made less entries and spent less time in the open arms of the EPM (Fig. 4E–G). In addition, we examined home-cage locomotor activity of offspring reared by *Clock* mutant mice. In a 24 hour period, total activity levels were not significantly different between offspring reared by wild-type and *Clock* mutant mice (Fig. 4H), suggesting that the alterations in behaviors in the paradigms did not simply reflect a decreased locomotor activity of the offspring. Thus, these observations suggest that offspring reared by *Clock* mutant mice display increased anxiety-related behavior. Similar increased anxiety-related behavior was also observed in offspring at 14–16 weeks of age (Figure S1).

We also performed Porsolt forced swim test and tail suspension test, in which the amount of time immobile in an inescapable situation is used as a measure of behavioral despair. These behavioral tests are often used in the context of studies on depression. In these tests, total duration of immobility was similar in offspring reared by wild-type and *Clock* mutant mice (Figure S2).

### Decrease in Serotonin Levels in the Brain of Offspring Reared by *Clock* Mutant Mice

**Figure 5:**
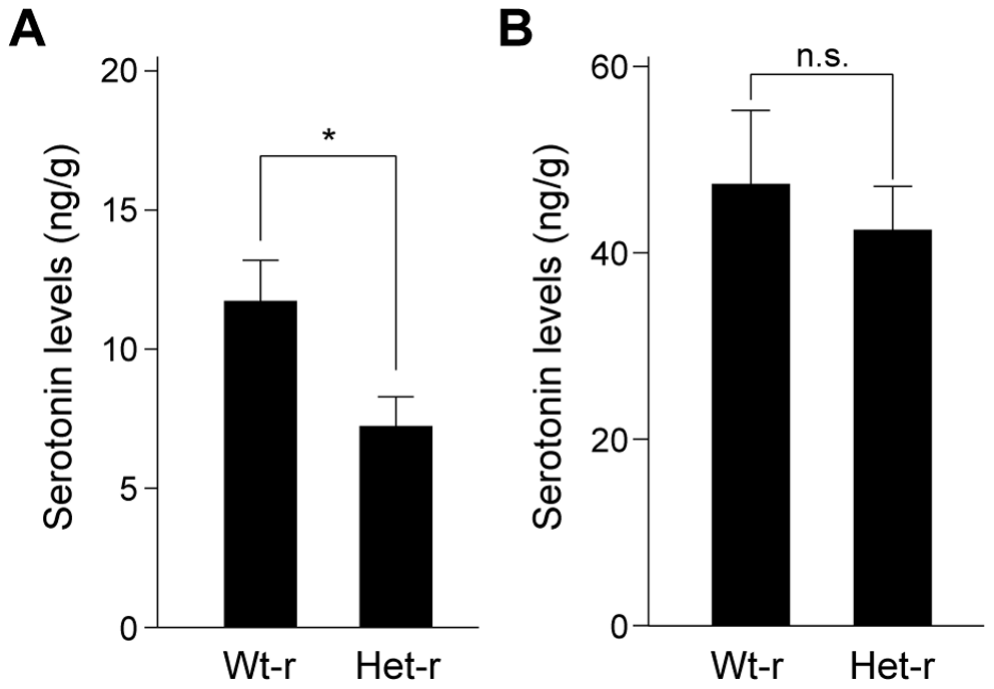
Brain serotonin levels in offspring reared by *Clock* mutant mice. (A, B) Serotonin levels in the brain of offspring reared by wild-type mice (Wt-r) and *Clock* mutant mice (Het-r) at 14 days old (A) and 7 weeks old (B). Data are presented as mean ± SEM (n = 5–8, n.s.: not significant, *p<0.05, Student’s *t* test). t(8) = 2.45, p<0.05 in (A), t(14) = 0.530, p>0.05 in (B).

Serotonin has been shown to be an important factor for the development of anxiety-modulating circuits, and alterations of serotonin levels and signaling during the early postnatal period lead to emotional disturbance in adulthood [6], [32]. We then sought to examine levels of serotonin in the brain of offspring. Offspring reared by *Clock* mutant mice had significantly lower levels of serotonin at postnatal day 14 than offspring reared by wild-type mice (Fig. 5A). On the other hand, no significant difference was observed in offspring at 7 weeks of age (Fig. 5B). These observations together suggest that rearing by *Clock* mutant mother leads to a decrease in serotonin levels in the brain of the offspring during the early development as well as predisposes the offspring to increased anxiety-related behavior in adulthood.

## Discussion

In the present study, we found that offspring reared by *Clock* mutant mothers exhibited increased anxiety-related behavior in adulthood. In humans, monkeys and rats, aversive and aberrant maternal care such as childhood abuse and maternal deprivation has been shown to increase the risk of development of emotional disturbance such as increased anxiety-related behaviors [3], [5], [10]–[16]. The present study suggests that impaired circadian rhythms in mothers can also become an aversive factor to predispose the offspring to increased anxiety-related behavior.

*Clock* mutant mother used in the present study (heterozygous *Clock* mutant mice) displayed altered daily pattern of nursing behavior without change in total duration of the behavior. This abnormality was less severe than homozygous *Clock* mutant mice that show arrhythmic nursing behavior with decreased total duration of the behavior [21]. In addition, homozygous *Clock* mutant mice show less anxiety-related behavior [33], whereas female heterozygous *Clock* mutant mice used in the present study showed no significant difference in the total activity and time spent at the center area in the OFT (Figure S3). We also found no significant difference in entries to and time spent in the open arms in the EPM (Figure S3). These results suggest that anxiety levels in female heterozygous *Clock* mutant mice were not significantly altered and that increased anxiety levels in the offspring reared by *Clock* mutant mice is unlikely attributable to alteration of anxiety levels in mothers. Considering the altered daily pattern of nursing behavior in *Clock* mutant mice, it is conceivable that appropriate temporal pattern of maternal care may be crucial for establishing normal anxiety levels in the offspring. It is known that quantity of licking/grooming of the mother affects anxiety levels of the offspring [7]–[9]. Increased anxiety levels of the offspring reared by *Clock* mutant mother is, however, unlikely attributable to the behavior, as evidenced by the absence of difference in magnitude of licking/grooming between wild-type and *Clock* mutant mothers (Figure S4). It is also hypothesized that certain milk constituents that affect maturation of anxiety levels of the offspring are altered in *Clock* mutant mice. Considering that brain serotonin levels at postnatal day 14 were reduced in the offspring reared by *Clock* mutant mother, *Clock* mutant mother might have lower levels of serotonin precursors in milk. To examine the possibility, we measured levels of tryptophan, a precursor of serotonin, in milk of *Clock* mutant mother. The levels of tryptophan in milk (as well as plasma in the pups) of *Clock* mutant mothers were not reduced. Rather, the tryptophan levels were increased (Figure S5), suggesting that decreased serotonin levels in the brain of the offspring reared by *Clock* mutant mothers are unlikely attributable to lower levels of tryptophan in milk of *Clock* mutant mothers. Nonetheless, it remains possible that some other maternal behaviors or milk constituents that are controlled by the circadian clock associate with increased anxiety levels of the offspring reared by *Clock* mutant mother. Together, appropriate circadian clock-controlled maternal cares/factors in mothers are likely to be important for the development of normal anxiety-modulating circuits in the offspring.

In the present study, we found that brain serotonin levels were altered in offspring reared by *Clock* mutant mice at postnatal day 14. Serotonin has been shown to be an important factor for development of anxiety-modulating circuits. In fact, functional genetic variants of serotonin transporter gene (5-HTT), which transports serotonin from the extracellular space, and the monoamine oxidase A gene (MAO-A), which is a key enzyme responsible for degrading serotonin, have been implicated in mental disorders such as mood and anxiety disorders [3], [5], [34]–[37]. It has also been reported that both insufficient and excessive levels of serotonin lead to increased anxiety-related behavior in animal models such as mice deficient to 5-HTT, MAO-A/B and tryptophan hydroxylase, an enzyme generating serotonin precursor 5-hydroxytryptophan [6], [32]. Noticeably, suppression of 5-HTT only in the early postnatal period (from postnatal day 4–21) mimics abnormal emotional behaviors seen in knock-out mice [38]. Also, serotonin 1A receptor expression during the early postnatal period, but not in the adult, has been shown to be necessary for the development of normal anxiety-related behavior in mice [39], [40], suggesting that disruption of serotonin homeostasis and signaling during the early postnatal period causes emotional abnormalities such as elevated anxiety-related behavior in adult. Thus, increased anxiety levels in offspring reared by *Clock* mutant mice are likely due, at least in part, to alteration of serotonin homeostasis and serotonergic system in the brain during the early development.

Modern 24-hour/7-day society and lifestyle such as chronic restriction of sleep, night work and rotating shift work inherently perturb our natural circadian behavioral patterns and our body’s circadian timing system [41]. Our study imply that irregular daily life schedule in parents may pose risks of emotional disturbance in their offspring, whose potential linkage has been noticed by several studies [42]–[45].

## Materials and Methods

### Ethics Statement

All animal experiments were conducted in accordance with guidelines set by The University of Tokyo and approved (permit number 21-01) by the Committee on Animal Care and Use of the Graduate School of Science in The University of Tokyo.

### Animals

Mice harboring a mutation in *Clock* (BALB/c background) were a kind gift from Joseph S. Takahashi (Northwestern University, Evanston, IL). Mice were housed under a 12 h light/12 h dark cycle with food and water available *ad libitum.* The room was kept at 23°C±1°C. Zeitgeber time (ZT) is used for representing biological time in light-dark cycles, in which ZT0 and ZT12 correspond to the lights-on time and the lights-off time, respectively.

For monitoring maternal behavior of mothers and performing behavioral tests of the offspring, 6–8 wild-type male neonates (BALB/c at postnatal day 1) were separated from their wild-type mother and fostered on postpartum wild-type mice or heterozygous *Clock* mutant mice, in the absence of male mice. The offspring were weaned at 28 days old and housed individually in a cage. The offspring at 7–16 weeks of age were subjected to four behavioral tests in the following order; open-field test, elevated plus maze test, forced swim test and tail suspension test. Mice were given one test per day for 4 consecutive days (see Fig. 2). For behavioral tests of female mice, animals (virgins) at 12–22 weeks of age were subjected to behavioral tests in the following order; open-field test and elevated plus maze test. Mice were given one test per day for 2 consecutive days.

### Evaluation of Locomotor Activity

Locomotor activity was recorded under a light-dark cycle by a video camera equipped with the infrared light. On the recorded video, the cage was horizontally divided into three equal parts by two lines, and the number of times that the mouse crossed the lines was counted. Activity counts in 5-min bins over a 24-hour cycle were measured for individual mice, and mean activity counts in 5-min bins were calculated.

### Evaluation of Nursing and Licking/grooming Behaviors

Maternal behavior was recorded under a light-dark cycle by a video camera equipped with the infrared light. Video-recordings were made on postpartum day 2–3. When mothers exhibit nursing postures (either an arched-back nursing posture, a blanket nursing posture in which mother lays over the pups, or a passive nursing posture in which mother is lying on her side with pups attached) for at least 5 min, we measured duration time of the nursing bouts.

We also measured duration time of licking/grooming behavior on postpartum day 2–3 in a 1-hour bin of 6 time-points within a day (ZT 2–3, ZT 6–7, ZT 10–11, ZT 14–15, ZT 18–19 and ZT 22–23). The duration time of licking/grooming at 6 time-points was then accumulated for individual mice and presented as percentage to the total observation time.

### Open Field Test

The apparatus consisted of a polypropylene white box (40×40×40 cm). The illumination at the level of the arena is 80–90 lx. The experiment was performed between ZT8–ZT12. For testing, mice were individually placed in one corner of the arena and allowed to explore the arena freely. The behavior of mice was video-recorded for 5 min. On the recorded video, the arena of the open filed was divided into 16 equal square areas; the four inner square areas in the center (center areas) and 12 squares in the periphery along the wall. Total number of squares crossed by the mouse, the number of center areas crossed, time spent at the center areas, and the percentage of the number of center areas crossed (100×center areas/total areas crossed) were measured for individual mice.

### Elevated Plus Maze Test

The apparatus consisted of four arms (30 cm long×5 cm wide) connected to the central platform (5 cm×5 cm). The maze was elevated 45 cm above the floor. Two of the arms were enclosed with walls (20 cm height, closed arms), and the other arms had no border in place of the walls (open arms). The illumination at the level of the platform is 60–70 lx. The experiment was performed between ZT8–ZT12. For testing, mice were individually placed on the central platform with its head facing toward a closed arm, and its behavior was video-recorded for 5 min. An entry was judged when all four paws of the mouse entered an open or closed arm. The number of open arm entries, the number of closed arm entries, time spent in the open arms, and the percentage of open arm entries (100×open arm/total entries) were measured for individual mice.

### Tail Suspension Test

Mice were individually suspended with the tail 8 cm above the floor in a grey box (21 cm wide×15 cm length×38 cm high) and video-recorded for 6 min. Immobility time was scored through 6 min duration of the test, as an index of behavioral despair in the face of an inescapable stress [46]. The illumination at the level of the mice was 900–1000 lx. The experiment was performed between ZT9–ZT12.

### Forced Swim Test

The apparatus consisted of a transparent glass cylinder (28 cm high×18 cm diameter) filled up to 15 cm with water that was equilibrated in the room temperature for more than 2 days. The illumination at the level of the floor was 1000–1100 lx. The experiment was performed between ZT9–ZT12. For testing, mice were gently placed in the water and video-recorded for 6 min. Total immobility time was measured for last 4 min, as an index of behavioral despair in the face of an inescapable stress [46].

### Measurement of Brain Serotonin

Animals were sacrificed by cervical dislocation at ZT16. The forebrain was separated from the brainstem at the level of superior colliculus, immediately frozen in liquid nitrogen and stored at −80°C until use. The brain samples were homogenized in 0.2 N perchloric acid (10 µl of 0.2 N perchloric acid/mg of tissue) by a Teflon-glass homogenizer. Thereafter, the homogenates were sonicated briefly (XL-2000 Microson Ultrasonic Cell Disruptor, Misonix, Level 5, 10 sec×2), centrifuged at 16,000×g for 30 min at 4°C. The supernatant was filtrated through 0.22 µm filter and neutralized with borate buffer. The resultant samples were used for measurement of serotonin with EIA serotonin kit (Beckman Coulter Company) according to the manufacturer's protocol.

### Measurement of Milk and Plasma Tryptophan

Mother mice on postpartum day 2–4 were separated from their fostering wild-type pups 2 hours prior to milking. Each mother was intraperitoneally injected with 0.1 ml (2 IU) of oxytocin. A second injection of oxytocin was carried out a few minutes later. Milk was collected into a 1.5 ml tube with a vacuum pump. Milk collection was performed at ZT4-6. Also, pups at postnatal day 14 were quickly decapitated at ZT16 for trunk blood collection. Blood was collected with Vacutainer EDTA tubes (Becton, Dickinson and Company). Blood samples were then centrifuged (1,200×g for 20 min at room temperature), and the supernatants (plasma extracts) were stored at −80°C until use. The milk and plasma samples were used for measurement of tryptophan with tryptophan ELISA kit (Labor Diagnostika Nord GmbH & Co. KG) according to the manufacturer's protocol.

### Statistical Analyses

All bar graphs were plotted as mean ± SEM. Data were analyzed by two-tailed Student’s *t* test. The significance level was set at p<0.05 for all tests. All statistical analyses were performed using Excel.

## Supporting Information

Figure S1 **Elevated anxiety-related behavior in offspring reared by** ***Clock*** **mutant mice.** Offspring reared by wild-type mice (Wt-r) or *Clock* mutant mice (Het-r) were subjected to behavioral tests at 14–16 weeks of age. In the open-field test, total activity counts (A), activity counts at the center area (B), time spent at the center area (C) and the percentage of the number of center squares crossed (D) are shown as in Fig. 4. Data are presented as mean ± SEM (n = 8, *p<0.05, Student’s *t* test). t(14) = 2.27, p<0.05 in (A), t(8) = 2.37, p<0.05 in (B), t(14) = 2.24, p<0.05 in (C), t(9) = 2.41, p<0.05 in (D). In the elevated plus maze test, time spent in the open arms (E), entries to the open arms (F) and the percentage of open arm entries (100×open arm/total entries) (G) are shown. Data are presented as mean ± SEM (n = 14–15, *p<0.05, Student’s *t* test). t(18) = 2.43, p<0.05 in (E), t(18) = 2.34, p<0.05 in (F), t(27) = 2.13, p<0.05 in (G).
(TIF)

Figure S2 **Forced swim test and tail suspension test of offspring reared by** ***Clock*** **mutant mice.** (A, B) Immobility time in the forced swim test (A) and in the tail suspension test (B) are shown as mean ± SEM (n = 6–13). No significant difference (n.s.: not significant, Student’s *t* test) was observed between offspring reared by wild-type mice (Wt-r) and *Clock* mutant mice (Het-r). t(19) = 1.05, p>0.05 in (A), t(16) = −0.213, p>0.05 in (B).
(TIF)

Figure S3 **Anxiety-related behavior in female** ***Clock*** **mutant mice.** Female wild-type mice (Wt) or *Clock* mutant mice (Het) were subjected to behavioral tests at 12–22 weeks of age. In the open-field test, total activity counts (A), activity counts at the center area (B), time spent at the center area (C) and the percentage of the number of center squares crossed (D) are shown as in Fig. 4. Data are presented as mean ± SEM (n = 7–8, n.s.: not significant, Student’s *t* test). t(13) = 1.71, p>0.05 in (A), t(13) = 0.561, p>0.05 in (B), t(13) = 0.205, p>0.05 in (C), t(13) = −0.532, p>0.05 in (D). In the elevated plus maze test, time spent in the open arms (E), entries to the open arms (F) and the percentage of open arm entries (100×open arm/total entries) (G) are shown. Data are presented as mean ± SEM (n = 7–8, n.s.: not significant, Student’s *t* test). t(13) = −0.358, p>0.05 in (E), t(13) = 0.422, p>0.05 in (F), t(13) = −0.0453, p>0.05 in (G).
(TIF)

Figure S4 **Licking/grooming behavior in** ***Clock*** **mutant mice.** Duration time of licking/grooming behavior on postpartum day 2–3 was measured in a 1-hour bin of 6 time-points within a day (ZT 2–3, ZT 6–7, ZT 10–11, ZT 14–15, ZT 18–19 and ZT 22–23). The duration time of licking/grooming at 6 time-points was then accumulated for individual mice and presented as percentage to the total observation time. Data are shown as mean ± SEM (n = 3). t(4) = −0.917, p>0.05 by Student’s *t* test. n.s.: not significant. Wt: wild-type mice, Het: heterozygous *Clock* mutant mice.
(TIF)

Figure S5 **Tryptophan levels in milk of** ***Clock*** **mutant mice.** (A) Tryptophan levels in milk of wild-type mother (Wt) and *Clock* mutant mother (Het) on postpartum day 2–4. Data are presented as mean ± SEM (n = 3–4). t(5) = −2.66, *p<0.05 by Student’s *t* test. (B) Plasma tryptophan levels in the offspring reared by wild-type mother (Wt-r) and *Clock* mutant mother (Het-r) at 14 days old. Data are presented as mean ± SEM (n = 3). t(4) = −3.78, *p<0.05 by Student’s *t* test.
(TIF)
