# Supplementary figures and images for: Increased Anxiety in Offspring Reared by Circadian Clock Mutant Mice
Source: PLoS One. 2013 Jun 12;8(6):e66021. doi: 10.1371/journal.pone.0066021 (PMC3680406; doi:10.1371/journal.pone.0066021)

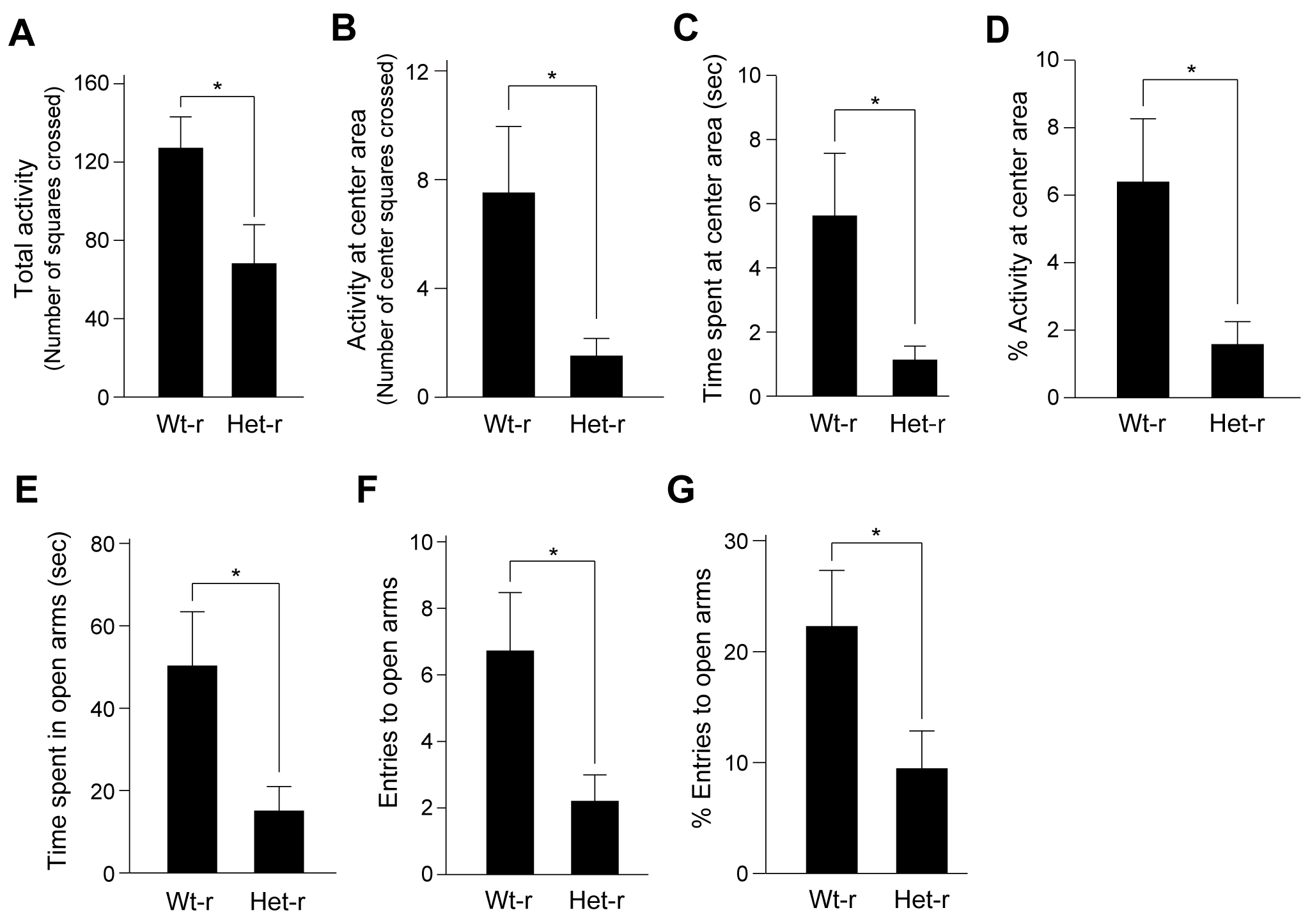

Supplement: Figure S1 — Elevated anxiety-related behavior in offspring reared by Clock mutant mice. Offspring reared by wild-type mice (Wt-r) or Clock mutant mice (Het-r) were subjected to behavioral tests at 14–16 weeks of age. In the open-field test, total activity counts (A), activity counts at the center area (B), time spent at the center area (C) and the percentage of the number of center squares crossed (D) are shown as in Fig. 4. Data are presented as mean ± SEM (n = 8, *p<0.05, Student’s t test). t(14) = 2.27, p<0.05 in (A), t(8) = 2.37, p<0.05 in (B), t(14) = 2.24, p<0.05 in (C), t(9) = 2.41, p<0.05 in (D). In the elevated plus maze test, time spent in the open arms (E), entries to the open arms (F) and the percentage of open arm entries (100×open arm/total entries) (G) are shown. Data are presented as mean ± SEM (n = 14–15, *p<0.05, Student’s t test). t(18) = 2.43, p<0.05 in (E), t(18) = 2.34, p<0.05 in (F), t(27) = 2.13, p<0.05 in (G). (TIF) [file pone.0066021.s001.tif]

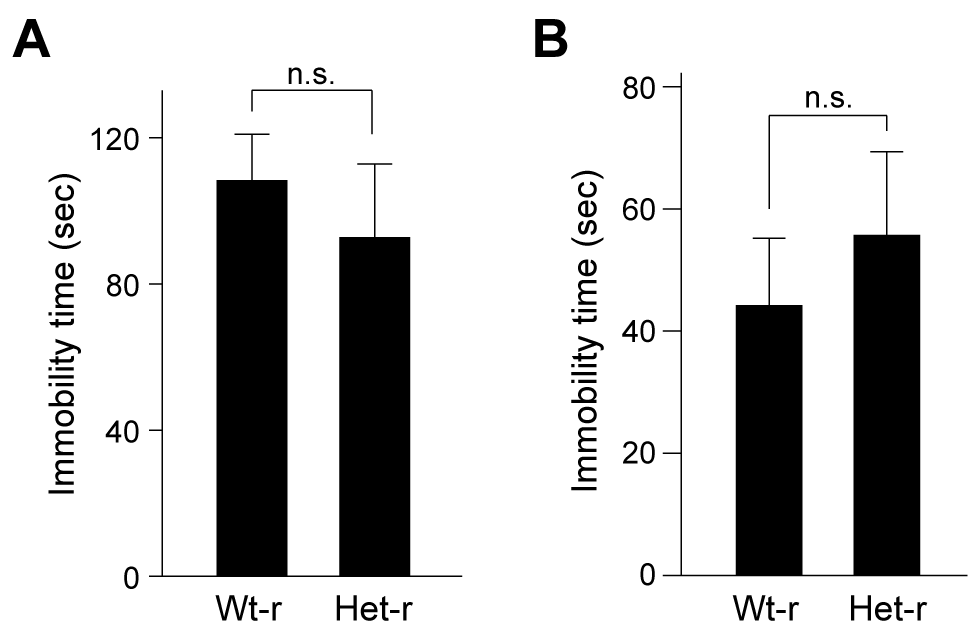

Supplement: Figure S2 — Forced swim test and tail suspension test of offspring reared by Clock mutant mice. (A, B) Immobility time in the forced swim test (A) and in the tail suspension test (B) are shown as mean ± SEM (n = 6–13). No significant difference (n.s.: not significant, Student’s t test) was observed between offspring reared by wild-type mice (Wt-r) and Clock mutant mice (Het-r). t(19) = 1.05, p>0.05 in (A), t(16) = −0.213, p>0.05 in (B). (TIF) [file pone.0066021.s002.tif]

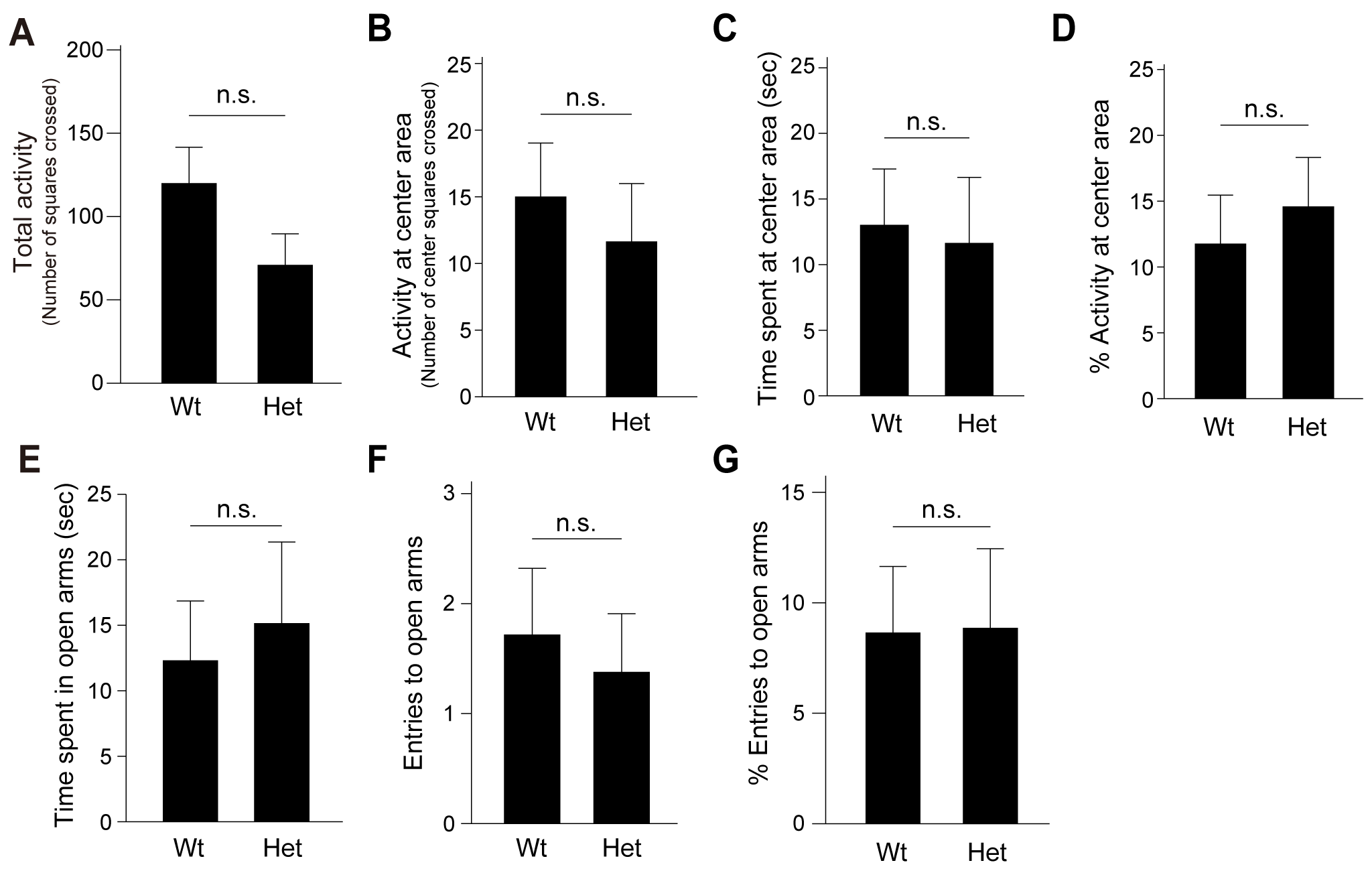

Supplement: Figure S3 — Anxiety-related behavior in female Clock mutant mice. Female wild-type mice (Wt) or Clock mutant mice (Het) were subjected to behavioral tests at 12–22 weeks of age. In the open-field test, total activity counts (A), activity counts at the center area (B), time spent at the center area (C) and the percentage of the number of center squares crossed (D) are shown as in Fig. 4. Data are presented as mean ± SEM (n = 7–8, n.s.: not significant, Student’s t test). t(13) = 1.71, p>0.05 in (A), t(13) = 0.561, p>0.05 in (B), t(13) = 0.205, p>0.05 in (C), t(13) = −0.532, p>0.05 in (D). In the elevated plus maze test, time spent in the open arms (E), entries to the open arms (F) and the percentage of open arm entries (100×open arm/total entries) (G) are shown. Data are presented as mean ± SEM (n = 7–8, n.s.: not significant, Student’s t test). t(13) = −0.358, p>0.05 in (E), t(13) = 0.422, p>0.05 in (F), t(13) = −0.0453, p>0.05 in (G). (TIF) [file pone.0066021.s003.tif]

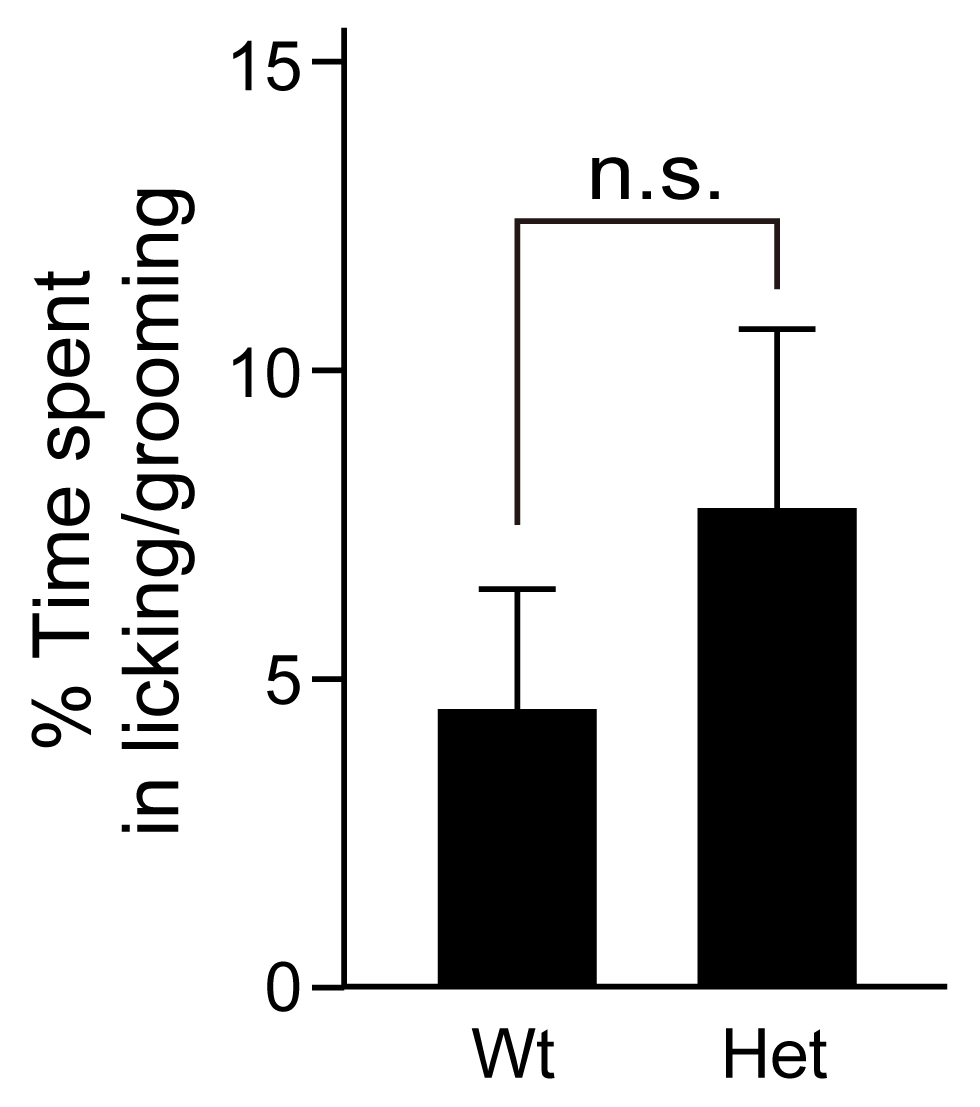

Supplement: Figure S4 — Licking/grooming behavior in Clock mutant mice. Duration time of licking/grooming behavior on postpartum day 2–3 was measured in a 1-hour bin of 6 time-points within a day (ZT 2–3, ZT 6–7, ZT 10–11, ZT 14–15, ZT 18–19 and ZT 22–23). The duration time of licking/grooming at 6 time-points was then accumulated for individual mice and presented as percentage to the total observation time. Data are shown as mean ± SEM (n = 3). t(4) = −0.917, p>0.05 by Student’s t test. n.s.: not significant. Wt: wild-type mice, Het: heterozygous Clock mutant mice. (TIF) [file pone.0066021.s004.tif]

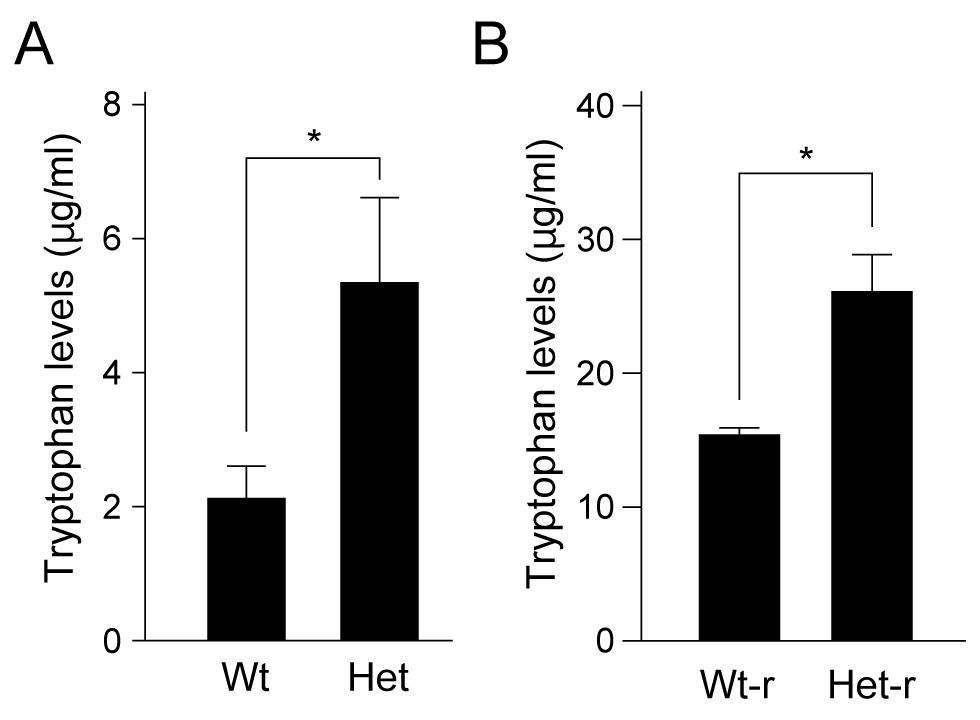

Supplement: Figure S5 — Tryptophan levels in milk of Clock mutant mice. (A) Tryptophan levels in milk of wild-type mother (Wt) and Clock mutant mother (Het) on postpartum day 2–4. Data are presented as mean ± SEM (n = 3–4). t(5) = −2.66, *p<0.05 by Student’s t test. (B) Plasma tryptophan levels in the offspring reared by wild-type mother (Wt-r) and Clock mutant mother (Het-r) at 14 days old. Data are presented as mean ± SEM (n = 3). t(4) = −3.78, *p<0.05 by Student’s t test. (TIF) [file pone.0066021.s005.tif]
